# Knowledge and awareness of policies and programmes to reduce adolescent pregnancy in Ghana: a qualitative study among key stakeholders

**DOI:** 10.1186/s12978-023-01672-2

**Published:** 2023-09-22

**Authors:** Bright Opoku Ahinkorah, Melissa Kang, Lin Perry, Fiona Brooks

**Affiliations:** 1 School of Public Health, Faculty of Health, University of Technology Sydney, Ultimo, NSW Australia; 2 Specialty of General Practice, Faculty of Medicine and Health, The University of Sydney, Sydney, Australia; 3 School of Nursing and Midwifery, Faculty of Health, University of Technology Sydney, Ultimo, NSW Australia; 4 Faculty of Health and Environmental Sciences/Te Ara Hauora Ā Pūtaiao, Auckland University of Technology, Auckland, New Zealand

**Keywords:** Adolescents, Ghana, Policy, Pregnancy, Programmes, Sexual and reproductive health

## Abstract

**Introduction:**

Adolescent sexual and reproductive health continues to be a major public health issue in low-and middle-income countries. While many countries have policies aimed at reducing adolescent pregnancy, evidence of their impact is unclear. This study sought to explore the knowledge and awareness of policies and programmes aimed at reducing adolescent pregnancy among health and education professionals and grassroot workers in Ghana.

**Methods:**

We employed a cross-sectional, qualitative study design involving semi-structured interviews with 30 key informants (health and education professionals and grassroot workers) in the Central Region of Ghana. We also conducted a desktop review of policies aimed at reducing adolescent pregnancy in Ghana. We used content analysis to analyse the data.

**Results:**

Eight of the 30 participants demonstrated awareness of policies aimed at reducing adolescent pregnancy but only two could elaborate on this. By contrast, 19 of the 30 participants were aware of relevant programmes and provided detailed description of their implementation and activities carried out under each programme. Despite participants’ low policy awareness and knowledge, their descriptions of the activities carried out under each programme aligned with the strategies and activities of the policies mentioned, as evident from the desktop review of the policies.

**Conclusion:**

Greater engagement of stakeholders in future policy development should increase policy awareness. Dissemination of policy content through community-based media channels and in local languages should promote and facilitate stakeholder engagement, which in turn should increase effective policy implementation with subsequent reduction of adolescent pregnancy.

**Supplementary Information:**

The online version contains supplementary material available at 10.1186/s12978-023-01672-2.

## Plain Language Summary

Adolescent pregnancy is a major public health issue in low-and middle-income countries. Efforts to reduce adolescent pregnancy have become major health priorities globally. Several international organisations, including the United Nations, have adopted strategies such as those featured under the Sustainable Development Goal (SDG) 3.7. These strategies seek to ensure universal access to sexual and reproductive healthcare services, including family planning, information and education, and the integration of reproductive health into national strategies and programmes by 2030. Many countries have policies and programmes with implementation strategies aimed at reducing adolescent pregnancy; however, evidence of their impact is unclear.

We explored the knowledge and awareness of policies and programmes aimed at reducing adolescent pregnancy among health and education professionals and grassroot workers in Ghana. We conducted interviews among 30 key informants in Ghana.

Few of the participants demonstrated awareness of policies aimed at reducing adolescent pregnancy and only two could elaborate on this. By contrast, most participants were aware of relevant programmes and provided detailed description of their implementation and activities carried out under them. Greater engagement of stakeholders in future policy development should increase policy awareness. Dissemination of policy content through community-based media channels and in local languages should promote and facilitate stakeholder engagement, which in turn should increase effective policy implementation with subsequent reduction of adolescent pregnancy.

**Supplementary Information:**

The online version contains supplementary material available at 10.1186/s12978-023-01672-2.

## Introduction

Sexual and reproductive health has been regarded as a key issue for young people since the 1994 International Conference on Population and Development [1]. Improvement in Adolescent Sexual and Reproductive Health (ASRH) subsequently became a major health priority globally [2], with a number of international organisations including the United Nations adopting strategies for its enhancement. These strategies include the Sustainable Development Goal (SDG) 3.7 which seeks to ensure universal access to sexual and reproductive healthcare services, including family planning, information and education, and the integration of reproductive health into national strategies and programmes by 2030 [3]. Nonetheless, adolescents in low-and-middle-income countries (LMICs) continue to experience poor Sexual and Reproductive Health (SRH) outcomes, including early pregnancy and childbearing [4, 5].

Adolescent pregnancy is usually associated with serious risks for both the mother and the foetus and can lead to intergenerational cycles of underemployment, low educational attainment, and poverty [6]. It remains a major public health issue in LMICs [7–9], where 95% of the 16 million global adolescent pregnancies occur [10]. Many countries within sub-Saharan Africa lead global rankings for rates of adolescent pregnancy [11]. In Ghana, in 2014, 14.2% of adolescent girls had begun childbearing: 11.3% had experienced a live birth, and 2.9% were pregnant with a first child [12]. These proportions are higher than the global estimate of adolescent birth rates which have decreased from 4.5% in 2000 to 4.25% in 2021 [10], reflecting ongoing challenges associated with the achievement of SDG 3.7 in LMICs such as Ghana.

There is strong international empirical evidence for the impact of high quality, comprehensive sex and relationships education linked to improved use of contraception for reducing rates of adolescent pregnancy [13]. Public health professionals, policymakers, and government and non-governmental organisations (NGOS) have implemented programmes focused on reducing adolescent pregnancy and promoting SRH policies [14].

In Ghana, national policies have aimed at reducing adolescent pregnancy [6, 15]. These policies have been useful in reducing adolescent pregnancy through mainstreaming SRH information and gender-sensitive and responsive health services [16]. Notable among these is the Adolescent Health Service Policy and Strategy which seeks to enhance the health status and quality of life of adolescents and young people in Ghana [17]. This policy, current at the time of writing, comprehensively addresses significant determinants of adolescent pregnancy such as access to family planning services, coerced sex, concurrent partners, child marriage, contraceptive use, early sexual initiation, multiple sexual partners and sexual violence [17, 18]. Other policies include the 2000 Adolescent Reproductive Health Policy, 2010 National Youth Policy, 2014 Child and Family Welfare Policy, and the 2015 National Gender Policy [6, 15].

In addition to national policies, the Ghana Health Service has also implemented the Ghana Adolescent Reproductive Health (GHARH) Project funded by the Palladium Group in selected regions. NGOs in the country have also implemented programmes aimed at reducing adolescent pregnancy [15]. For instance, a child rights organization (Afrikids) in collaboration with the Ghana Health Service and other stakeholders in the Upper East Region commenced a project to promote sex education in seven districts to reduce high teenage pregnancy and unsafe abortion rates in the region in 2016. The GOSANET Foundation also implemented a project in the Adaklu District of the Volta region of Ghana with an overall goal of improving and advancing the SRH rights of in- and out-of-school adolescents through the provision of appropriate education and linkage to access services. These programmes promote access to SRH services, including family planning. The implementation of these programmes began in 1994 when the Population Policy of Ghana was revised and there was a recognised requirement for programmes to address the specific reproductive health needs of young people in line with the revised policy [15].

Knowledge and awareness are essential prerequisites and precursors of action and there is evidence that increased knowledge and awareness can lead to better implementation of policies and programmes [19], which may ultimately make these policies and programmes more effective and lead to improved SRH among adolescents [20]. However, to date, no study in sub-Saharan Africa has explored the awareness and knowledge of these policies and programmes among those responsible for implementing their related strategies and activities. Few distantly related studies exist which explored the views of stakeholders on policy positions, attitudes and involvements of government/public institutions and NGOs in ASRH policy-making processes and implementations in Ebonyi State, Nigeria [21], and examined the current knowledge, behaviours and attitudes towards sexual and reproductive health and rights among adolescent girls and young women, service providers and stakeholders [22]. None of them specifically focused on knowledge and awareness of policies and programmes aimed at reducing adolescent pregnancy. We, therefore, sought to explore the knowledge and awareness of policies and programmes aimed at reducing adolescent pregnancy among health and education professionals and grassroot workers, using Ghana as an example.

## Methods

### Design

With approval from the Ghana Health Service Ethics Review Committee and the Human Research Ethics Committee of the University of Technology Sydney, we employed a qualitative study design involving semi-structured interviews with 30 key informants (health and education professionals and grassroot workers). A realist approach to implementation was applied in this study [23], focusing on examining what interventions work with this specific population and environment. We also conducted a desktop review of policies aimed at reducing adolescent pregnancy in Ghana in order to verify information provided by interview participants and to map the policies they named to relevant and current national policies. This study forms part of a larger doctoral study conducted by the first author.

### Study setting

The study setting was communities, government and non-government organisations within three districts in the Central Region of Ghana: Komenda-Edina-Eguafo-Abrem municipality, Cape Coast municipality, and Assin South district in the Central region of Ghana. This region has higher rates of adolescent pregnancy (7.0%) compared to the national average (2.9%) [12, 24–27]. Approximately 78% of the population of Central Region are literate. This is higher than the national average of 74.1 percent, but falls below the rates of three other Regions- Greater Accra (89.3%), Ashanti (82.6%) and Eastern Region (81.0%). Rural–urban variations in literacy levels exist in the region, with 82% proportion literate in urban localities compared with 74.6% in rural areas. The districts share similar demographics: fishing and farming are the major economic activities, Fante and Twi are the dominant languages and most people are Christian. The districts are relatively rural and most residents are of low socio-economic status [28].

### Study sample and sampling technique

Our study sample was made up of 15 health and education professionals and 15 grassroot workers. All the 15 health and education professionals work across the three districts in the region. These districts were selected because they have higher reported rates of adolescent pregnancy compared with the national average [26]. Five grassroot workers were selected from each of the three districts. To develop the sampling frame for the health and education professionals, we consulted the heads of the regional health and education directorates and the directorate for gender. The health and education professionals included the regional director of the Department of Gender, regional School Health Education Programme (SHEP) coordinator and programme coordinators for NGOs that offer ASRH services in the region. We also retrieved some names of health and education professionals from the internet and through recommendations from other health and education professionals. The regional director of the Department of Gender, who oversees the government’s women’s rights and empowerment portfolio under which adolescent sexual and reproductive health sits, helped us locate some of the health and education professionals. The grassroot workers included chiefs, queen mothers (traditional female leaders, drawn from the relevant royal lineages, who are mostly responsible for women's and children's issues), youth leaders and community health volunteers. They played roles such as that of assembly members, queen mothers, facilitators of NGOs, chief’s linguists, parent-teacher association chairs, youth leaders, committee members and secretaries. A list of these individuals was obtained from the three districts in the region through consultation with heads of NGOs and health and education professionals who were involved in the implementation of programmes aimed at reducing adolescent pregnancy in the districts.

### Data collection procedure

#### Instrument

An interview guide was the data collection instrument. This was developed by the authors, based on international literature about sexual and reproductive health policies and programmes [18]. Interview prompts included awareness, knowledge, and descriptions of national and/ or district level policies and programmes as well as the participant’s role in implementation (Additional file 1). For this study, where a participant mentioned a policy or programme, this was taken to indicate they were aware of and had some knowledge of it. The instrument was piloted with two health and education professionals in Accra who had worked across several districts in the Central region.

#### Interviews

Individual interviews were conducted face-to-face and via WhatsApp™ using mobile phones by two trained research assistants. Interviews were conducted between 20th August and 9th November 2020. During the interviews, health and education professionals were asked if they knew of any policies and programmes. Those who could mention any policies and programmes were asked further to describe those policies and programmes by mentioning some of their key strategies/activities. Grassroot workers were only asked to mention any programmes. Those who could mention any of these were probed further to describe the programmes. Face-to-face interviews took place in community buildings, private offices or homes of participants, based on participants' preference. Interviews with health and education professionals were conducted in English while those with grassroot workers were conducted in either Fante or Twi. The interviews lasted between 45 and 120 min. Interviews were audio-recorded and professionally transcribed; those conducted in Fante and Twi were professionally translated. Field notes were taken in addition to the recordings. Informed consent was obtained from all participants after they had been fully informed about the aim of the study, its procedures and all possible risks.

### Data analysis

English language transcripts were analysed. We used NVivo version 12 to assist with data organisation and analysis. The first author read and coded all transcripts and the second author read and independently coded 20% of the transcripts; all authors read and commented.

First, codes were inserted as comments in a MS Word file. These files were then imported into NVivo version 12 to assist with data organisation and analysis. In NVivo, all thirty transcripts were coded again by the first author. Coding was done inductively. Content analysis was used to enumerate policies and programmes mentioned by participants, and to create text descriptions based on what the participants said.

#### Desktop review

To ensure accuracy of the policies mentioned by the participants, we carried out a desktop review of ARSH policies in Ghana, seeking to identify those mentioned by participants. This verified the currency of the policies and provided detailed description of their goals and strategies. 

A Google™ search of policies aimed at reducing adolescent pregnancy in Ghana was conducted and relevant policies retrieved. The content of the policies was reviewed and the key strategies focused on reducing adolescent pregnancy were enumerated in a table.

### Research credibility and trustworthiness

To ensure credibility, debriefing was ensured through constant dialogue with the co-authors via e-mail and Microsoft Teams. De-identified transcripts of interviews were sent to the co-authors on a secured server so they could read and make comment. Making field notes, keeping recorded data and interview notes and documenting decisions related to data analysis such as coding, themes and categories enhanced the trustworthiness of the data. The findings that emerged from the data further contributed to the trustworthiness of the data. The engagement of the co-authors allowed for multiple perspectives of the data and analysis.

## Results

### Description of study participants

Thirty key informants were interviewed. Seventeen health and education professionals were invited, but two were not included because their supervisors did not return approvals in time. Of the fifteen health and education professional participants, eight were female, seven male and their ages ranged from 21 to 50 years. They worked across the three districts in the central region for government and non-governmental organisations including the United Nations Population Fund (UNFPA), Planned Parenthood Association of Ghana (PPAG), International Needs Ghana, National Youth Authority, Ghana Education Service, Curious Minds, Progressive Excellence Youth Organisation, Ghana Health Service, PURIM Africa Development Network, Department of Gender, and Marie Stopes International.

All invited grassroot workers consented to participate and were interviewed. They comprised ten males and five females, aged between 28 and 55 years. Five grassroot workers were selected from each of the three districts. The grassroot workers included queen mothers, chiefs, linguists, assemblymen and other community members who served as key contact persons in the implementation of sexual and reproductive health programmes.

### Policies

Eight of the fifteen health and education professionals demonstrated awareness of a total of seven current population health policies which were verified by desk review. Two of the eight health and education professionals (with 16 and 20 years of work experience) provided details about specific strategies such as access to reproductive health for adolescents, sex education for adolescent girls, and advocacy among stakeholder organisations within policies. These eight participants had an average of 11 years of experience compared to eight years for those who could not demonstrate awareness of any policies. Two other policies (described as related to provision of free senior high school education and educational re-entry for girls after teenage pregnancy) were mentioned by the health and education professionals and six of the grassroots workers but these could not be verified from the desk review and hence were excluded from further consideration. The seven policies referred to by the participants are listed in Table 1, with information from the desktop review and the number of participants who demonstrated awareness of each. These are set out using illustrative quotations from participants to describe the purpose, strategies and/or activities within each policy, as they understood this.

**Table 1:** Policies aimed at reducing adolescent pregnancy in Ghana identified by health and education professionals

Policies	Key strategies as stated in the policy	Number of participants naming each policy; (n = 15)
Adolescent Health Service Policy and Strategy, (2016–2020) [17]	• Using social and behavioural change communication strategies to enhance adolescent sexual and reproductive health • Improving access to family planning services among adolescents • Reducing school drop-out rates • Preventing and responding to harmful practices such as forced marriages and sexual violence against adolescents	1
Adolescent Reproductive Health Policy, 2000 [29]	• Adolescent sexual and reproductive health education using mass media, counselling, symposia and club activities • Strengthening the teaching and learning of reproductive health issues in schools and organizing reproductive health programmes for out of school adolescents • Promoting pre-marital sexual abstinence as an acceptable way of life • Establishment of youth centres/libraries to provide adolescents with sexual and reproductive health services • Enhancing youth involvement in the formulation and implementation of sexual and reproductive health services • Involving youth in the formulation and implementation of sexual and reproductive health services • Increasing availability of and accessibility to adolescent reproductive health services, including family planning	4
Child and Family Welfare Policy, 2014 [30]	• Economic empowerment through Livelihood Empowerment Against Poverty (LEAP), capitation grants, the National Health Insurance Scheme and free maternal care, school uniforms or school feeding programme • Youth involvement in decision making processes • Research, monitoring and assessments of child protection issues	2
Five Year Strategic Plan to Address Adolescent Pregnancy in Ghana, (2018–2022) [31]	• Empowering adolescents to make choices regarding their sexual debut and enabling them to prevent early and unplanned pregnancies • Promoting institutional and community engagement to prevent adolescent pregnancy • Ensuring that adolescents, especially those who are sexually active have access to youth-friendly and gender-responsive sexual and reproductive health information and services • Expanding adolescents' access to education and retention beyond Junior High School level especially for girls	2
National Gender Policy, 2015 [32]	• Promoting educational and issue–related programmes for total elimination of harmful practices including child marriages • Enforcing the teaching of age-appropriate education to girls and boys on sexuality and reproductive health and rights in school curricula, including issues of gender relations and responsible sexual behaviour, focused on preventing teenage pregnancies • Developing and implementing scholarship schemes for girl children and ensuring girls are retained in school to complete and move on to the next levels to avoid being victims of child and early marriage and motherhood situations that disempower them	2
National Strategic Framework to end Child Marriage, (2017–2026) [33]	• Empowering girls and boys to be better able to prevent and respond to child marriage • Influencing positive change in communities’ beliefs and attitudes and social norms that drive child marriage • Accelerating access to quality education, sexual and reproductive health information and services and other opportunities • Ensuring national laws, policy frameworks and mechanisms related to ending child marriage are in place, effectively enforced and implemented	2
National Youth Policy, 2010 [34]	• Improving the knowledge of youth about preventive health care and assisting them avoid practices such as engaging in early and irresponsible sexual activities • Developing programmes that will keep pupils and students in school to reduce school drop-outs • Providing apprenticeship training for out -of -school youth • Mentoring youth through the use of role models	3

### Adolescent Health Service Policy and Strategy

Of the 15 health and education professionals (HEPs), only one mentioned this as a policy aimed at reducing adolescent pregnancy in Ghana. This participant only mentioned that the country has this policy but could not elaborate on it.

When you look at Ghana, we have developed the Health Service Policy and Strategy for adolescent and youth health from 2016 to 2020. That is the policy document that is being used (HEP 9, Male, 36 years).

A review of this policy showed a number of strategies aimed at reducing adolescent pregnancy. The policy lists four specific strategies for reducing adolescent pregnancy (Table 1). The participants did not mention any of these strategies.

### Adolescent Reproductive Health Policy

Four health and education professionals demonstrated awareness of this policy. They described it as a policy that focuses on the reproductive health issues of adolescents and aims to provide adolescents, especially girls, with the information they need to make informed choices. The policy focused on SRH education in schools, including making adolescents aware of access to family planning services. One participant provided further detail about this policy, emphasising the role of access to contraceptives:

One of the key things in that policy to me is the fact that a woman can access contraceptives without necessarily the consent or permission of the husband or partner. Again, in that same policy, for adolescents who are sexually active, the policy allows them to access contraceptives (HEP 4, Male, 30–35 years).

A desktop review of this policy listed seven specific strategies for reducing adolescent pregnancy (Table 1). Two of these were mentioned by HEP 4 (increasing availability of and accessibility to adolescent reproductive health services, including family planning).

### Child and Family Welfare Policy

Two professionals cited the Child and Family Welfare Policy, with one able to provide a brief description of one of its goals:

We also have Child and Family Welfare Policy, which seeks to protect young people, particularly girls from some of the ills of society (HEP 13, Female, 40–45 years).

Desktop review showed three specific strategies aimed at reducing adolescent pregnancy in Ghana (Table 1). None of these strategies were mentioned by the participants.

### ‘Five year strategic plan to address adolescent pregnancy in Ghana

Two participants mentioned this policy. Only one was able to elaborate in detail:

When you take the adolescent pregnancy strategic plan for instance, it is looking at issues such as access to reproductive health for adolescents, sex education for adolescent girls, and advocacy among stakeholder organisations and individuals like the traditional authorities, state institutions and all these people. So all these go into it (HEP 13, Female, 40–45 years).

Desktop review showed four strategies to reduce adolescent pregnancy (Table 1). The detailed description of the policy by HEP 13 aligns with three of the policy’s strategies: empowering adolescents to make choices regarding their sexual debut and enable them to prevent early and unplanned pregnancies; promoting institutional and community engagement to prevent adolescent pregnancy; and ensuring that adolescents, especially those who are sexually active, have access to youth-friendly and gender-responsive sexual and reproductive health information and services.

### National Gender Policy

Two of the participants demonstrated awareness of this policy. However, only one provided further details.

First of all, as a country we have a National Gender Policy. The policy is about gender equality and the empowerment of girls. So, it is gender equality and empowerment of women and girls, and of course, adolescent reproductive health and rights are a part of our activities (HEP 13, Female, 40–45 years).

Desktop review showed three key strategies to reduce adolescent pregnancy (Table 1). HEP 13 mentioned two of these strategies (empowerment and gender equality).

### National Strategic Framework to End Child Marriage

Two of the health and education professionals also mentioned this policy. The participants described it as an ongoing national strategic framework which has been implemented to deal with child marriage in the country.

We also have the National Strategic Framework to End Child Marriage. It is something which is ongoing at the national level and aims at reducing child marriage (HEP 3, Female, 25–29 years).

### National Youth Policy

Three health and education professionals demonstrated awareness of the National Youth Policy. One described it as a multi-faceted policy:

“The national youth policy has a number of thematic areas for organizing programmes for the youth.” (HEP 4, Male, 38 years).

Another participant elaborated on health, wellbeing and education:

Health, wellbeing, and education are the thematic areas of the National Youth Policy. Under the health and wellbeing, it is aimed at promoting universal health coverage, increase health delivery, healthy lifestyle and physical wellbeing of the youth (HEP 14, Female, 25–29 years).

The National Youth Policy lists four key strategies aimed at reducing adolescent pregnancy (Table 1) and the description of the policy by HEP 14 aligned with the strategy on preventive health care.

### Programmes

Health and education professionals and grassroot workers mentioned twelve programmes which aim to reduce adolescent pregnancy (see Table 2).

**Table 2:** Awareness of programmes aimed at reducing adolescent pregnancy in Ghana among health and education professionals and grassroot workers

Programmes	Number of health and education professionals who mentioned each programme	Number of grassroots workers who mentioned each programme
Community Breaking the Silence	3	0
Community Parents Network Advocacy Group (COPNAG)	2	3
Community Sexual and Reproductive Health Outreach Programmes	5	0
Comprehensive Sexuality Education Programme	6	0
Livelihood Empowerment against Poverty (LEAP) programme	0	2
Livelihood or Vocational Training for Adolescent Girls	12	7
Promoting Safe Space for Adolescents (PASS) programme	1	4
Promotion, Empowerment and Community Action against Child Marriage	3	1
Safety Net Programme	2	0
School Feeding Programme	0	2
Time with Grandma	4	2
Youth Mentorship Programmes	7	2

### Community breaking the silence

Three health and education professionals but none of the grassroot workers mentioned and described the Community breaking the silence programme. This programme is organised by the Planned Parenthood Association of Ghana (PPAG) in collaboration with other stakeholders and is aimed at identifying sexual and reproductive health challenges within communities through a meeting with community leaders, parents and adolescents. One health and education professional described the programme as follows:

We are working hand-in-hand with Domestic Violence and Victims Support Unit (DOVVSU), Ghana Health Service and social welfare. These people come and educate the youth, parents, and opinion leaders in the community on adolescent pregnancy. After that, the youth, parents, and opinion leaders bring out bye-laws that can help prevent adolescent pregnancy (HEP 2, Female, 21 years).

Another health and education professional also confirmed the community breaking the silence programme as one of the programmes organised by the PPAG with the aim of bringing together community leaders and adolescents to identify sexual and reproductive health challenges within communities and find solutions. He explained the programme as follows:

Last week, I went to PPAG and they had this programme, where they will bring the opinion leaders and the youth together to find a way to solve their problems, then the chiefs and opinion leaders will know how best they will solve the problems (HEP 5, Male, 38 years).

### Community Parents Network Advocacy Group (COPNAG)

Two health and education professionals and three grassroot workers mentioned and described COPNAG. This was described as a programme implemented by the Department of Gender, aimed at empowering parents through sexual and reproductive health education so they can educate their children. COPNAG was described as educating both parents and adolescents on adolescent pregnancy and how they can work together to reduce it in their communities. Through the programme, parents are able to support adolescents to make informed decisions about their sexual health.

We stand for the education of adolescent girls so as to reduce teenage pregnancy and also the education of parents so that they can perform their duties by educating the girls to help reduce teenage pregnancy (GW 12, Male).

We started the Community Parent Networking Advocacy Group so that parents would understand issues of adolescents, the challenges of adolescents, how to provide education for their children and even how to support their girl child so that they don’t even get into that situation (HEP 13, Female, 40–45 years).

### Community sexual and reproductive health outreach programmes

Six health and education professionals described these as programmes organised by some non-governmental organisations, especially the PPAG, to make sexual and reproductive health services such as contraceptives accessible to adolescents through outreach. In most instances, these services were provided for free. The community sexual and reproductive health outreach programmes were described as follows:

The next strategy we employ is community outreach. For us, when we go for outreaches, our services are given for free so that people will be motivated to come because cost is another impediment aside distance and access (HEP 4, Male, 30–40 years).

Yes, especially PPAG. You know, they have a mobile van. Sometimes, they even come to our programmes and talk about contraceptives. They even provide free contraceptives to the youth who are interested (HEP 5, Male, 38 years).

### Comprehensive sexuality education (CSE) programme

Five health and education professionals but none of the grassroot workers mentioned the CSE programme, which has been modified as Reproductive Health and Education Services for Youth or young people (RHESY). Those who mentioned it described it as a programme to provide comprehensive sexuality education including sexual and gender-based violence, pregnancy and pregnancy-related issues, substance abuse, contraceptives, comprehensive abortion, and sexually transmitted infections.

Currently, what we seek to do is to make sexuality education comprehensive. We have sexual and gender-based violence, pregnancy and pregnancy-related issues, substance abuse, contraceptives, comprehensive abortion, sexually transmitted infections and so on (HEP 4, Male, 30–40 years).

One of the participants mentioned that major elements of the programme included education on personal hygiene and all other issues that are peculiar to girl children.

We have RHESY sessions. They meet the young people, educate them from basic knowledge to personal hygiene and anything related to the female child. (HEP 2, Female, 21 years).

### Livelihood empowerment against poverty (LEAP) programme

Two grassroot workers but no health and education professional mentioned the LEAP programme. LEAP was described as providing financial support to children and orphans in need. Through the programme, adolescent girls, who hitherto would have had sex with males because of financial challenges, have stopped that practice.

… prior to the LEAP, parents could become so financially constrained that when the child goes to the parent for money, the mother (does not have it). So the child will find a guy who has money and then have sex with them for money (GW 2, Male, 42 years).

When they pay the money to you, then you must work with it so that you can earn more to take care of the orphans and their daily upkeep. So, that is what the LEAP does (GW 7, Male, 55 years).

### Livelihood or vocational training for adolescent girls

Mentioned by twelve of the health and education professionals and seven of the fifteen grassroot workers, this programme was described as providing skills for girls to generate income through self-employment. Examples provided included teaching girls to make soap, detergents, cosmetics and beads; training them in baking and hairdressing. They also learned business skills and were able to be self-employed. The income earned empowered them and enabled them to avoid financial dependence on men:

Some of these girls have been able to start something for themselves. So they are now ending their relationships. So we are using those things to reduce teenage pregnancy (HEP 1, Male, 49 years).

Also, they train the girls on vocational skills so that the girls will be empowered and not be lured into going to have sex with a man since he is the one taking care of you. (GW 6, Male).

### Promoting safe space for adolescents (PASS) programme

One health and education professional and four grassroot workers described the PASS project, which was implemented by the NGO International Needs Ghana. The aim is to enable adolescent girls to live freely and be empowered to notice and identify threats to their sexual and reproductive health. These include their ability to discuss sexual and reproductive health issues freely in their homes, ability to refuse their partners sex and ability to detect the likelihood of sexual violence situations. The programme was described as follows:

PASS –… educates girls about the best ways to protect themselves and also how they can read the intentions of men or boys who come to propose to them (GW 2, Male, 48 years).

…provide(s) a safe space where adolescent girls in particular can meet to discuss issues to have a solution to it (HEP 13, Female, 40–45 years).

### Promotion, empowerment and community action against child marriage (PECACEM)

Only one health and education professional mentioned PECACEM by name but other health and education professionals and grassroot workers described similar programmes. One participant explained that PECACEM has led to the implementation of community guidelines on ending child marriage in several communities within the Central region of Ghana. These guidelines contain practical approaches in dealing with child marriage. These include sex education and dealing with sexual abuse in communities. She described these community guidelines as follows:

In the Central region specifically, we have the community guidelines on ending child marriage, sexual and domestic violence and promoting sex education. It gives information on how to provide sex education. Then also, it also gives information on what to do when there is sexual abuse. Apart from that, it also provides information on state institutions that a community leader can contact in the event of some of these issues (HEP 13, Female, 40–45 years).

### Safety net programme

Two health and education professionals but no grassroot worker mentioned this programme which was described as targeting pregnant adolescents to assist them during pregnancy, delivery and postnatal periods. They also provide family planning services after delivery to prevent subsequent pregnancies. One of the participants described the Safety Net Programme as follows:

The Safety Net Programme is tailored to meet the needs of pregnant adolescents. During their pregnancy and during our contact with them, we start education on family planning for them so that by the time they are ready to deliver, they would have decided on which family planning commodity they will go in for. Immediately they deliver, we do the family planning for them so that they don’t go and get pregnant again but rather focus on their after-pregnancy plan like focusing on their education and returning to school or learning a trade (HEP 9, Male, 29 years).

### School feeding programme

Two grassroot workers but no health and education professional mentioned the school feeding programme. The programme was described as helping to reduce adolescent pregnancy through poverty reduction. Poverty was described as contributing to adolescent pregnancy. Hence, adolescents who are fed in school through the school feeding programme will not go hungry and have sex with men to get money to buy food. They described the programme as follows:

Through the programme, children are given free food in school and this has reduced the financial burden on parents. Also, it has helped girls to stop asking for money from men and that has helped to reduce the chances of them having sex for money and end up pregnant (GW 2, Male, 42 years).

Also at school, they are given free meals so that the adolescents cannot say that it was because of such constraints that they engaged in sexual activities and got pregnant (GW 5, Female, 34 years).

### Time with grandma

Four health and education professionals and two grassroot workers mentioned this programme. These participants described it as a mentorship programme initiated by the Ghana Health Service, where adults in communities took on discussion of previously taboo sexual health issues, mentoring children/adolescents through story telling. Another participant, who worked with the Ghana Health Service, described its purpose as to instil morality in girls, enhancing their sexual and reproductive health and their ability to take control of this for themselves.

They select an adult from the community with good character who mentors the children through story telling. So based on that, the children are able to go to school and attain what they want to attain. Now we have grandpas and grandmas who are used as models who use storytelling to educate the adolescents (HEP 1, Male, 49 years).

We reach out to young people in the community and engage them so that they can be assertive, have confidence and then be able to take control over their life so that they can be able to navigate through this transition (HEP 9, Male, 36 years).

### Youth mentorship programmes

Seven health and education professionals and two grassroot workers mentioned youth mentorship programmes. These are organised through youth groups, adolescent community clubs, adolescent health clubs and annual conferences for adolescents. Hence, adolescents or youth who belong to these groups and those who attend the conferences are empowered through education on sexual and reproductive health and this makes it possible for them to make informed decisions about their sexual and reproductive health. They described the youth mentorship as follows:

We have formed adolescent groups in majority of our communities so that young people are educated on a daily basis on the dangers of early initiation of sex and having multiple sexual partners so that they can prevent some of these things (HEP 9, Male, 36 years).

Now, there is annual conference for adolescents which is organized by the Ghana Health Service and Population Council. They have formed adolescent groups in districts and they meet at the national level. They also have a board which is made up of adolescents where they discuss issues about themselves. Now, adolescents are being engaged at all levels (HEP 4, Male, 30–40 years).

## Discussion

To the best of our knowledge, this is the first study to have explored the awareness and knowledge of health and education professionals and grassroot workers in Ghana on policies and programmes aimed at reducing adolescent pregnancy. We found that few participants could demonstrate awareness of relevant policies and only two could provide any description, of two policies. By contrast, most participants were able to demonstrate awareness of a number of programmes. Despite participants’ low policy awareness and knowledge, their descriptions of the activities carried out under each programme aligned with the strategies and activities of the policies mentioned, as evident from the desktop review of the policies. This suggests that the programmes being implemented were aligned with policy, at least to some extent.

Several factors might account for the reported low policy awareness and knowledge despite that we purposively sampled professionals who were considered to have in-depth knowledge on the implementation of policies and programmes on adolescent pregnancy in Ghana. First, in most instances, policy development and implementation have been considered distinct and separate stages within policy cycles in Ghana [35, 36]. In this regard, not all key stakeholders for the implementation of policies are involved in policy formulation [37, 38]. Moreover, there is often inadequate policy dissemination among the key stakeholders for policy implementation. For instance, a study of the experiences of key national and sub-national health stakeholders to implement a national non-communicable diseases policy in Ghana showed that most reported low awareness of the non-communicable diseases policy, which was attributed to its poor dissemination [39]. The dissemination of policies should target all stakeholders, including grassroot workers, who are seldom directly involved in policy formulation, including in LMICs such as Ghana [40].

Seven policies were mentioned by the health professionals, and perhaps their awareness was because these professionals were either involved in their development or implementation. To ensure the effectiveness of policies in addressing adolescent pregnancy, it is essential that these and other key stakeholders have knowledge and awareness of the policies which they have responsibility to implement. Such knowledge and awareness can be acquired through involvement in the development of the policies. Simultaneously, policy-making processes and the policies produced benefit by the input of people with in-depth experience of the situation and issues addressed, and who understand and will be responsible for policy implementation.

Lack of grassroot worker involvement in policy formulation likely reflects a top-down approach to policy development and implementation [41, 42], and Ghana’s experience would be no different to that of many countries in this regard [43]. Politicians and government officials are directly involved in decisions regarding the development of policies at the national level. Government officials of various government and non-governmental organisations are responsible for the implementation of policies’ strategies and activities at the regional and district levels. At the operational level, grassroot workers are responsible for policy implementation through community-based programmes and activities [41]. Hence, grassroot workers may only be aware of activities they are delivering through training and workshops provided to support this, and may not know what policies guide these activities, as evidenced in a study conducted in Malaysia and Zanzibar [43]. Notwithstanding, it is essential that grassroot workers are aware and have knowledge of the policies that are driving programmes since their involvement is likely to strengthen policies and ultimately improve the reproductive health of adolescents in Ghana through improved programming. These grassroot workers mostly live in communities with adolescent girls and their understanding of context-specific factors could enhance the targeting and effectiveness of policies.

Most participants were aware of and had adequate knowledge of several programmes aimed at reducing adolescent pregnancy in Ghana. The detailed description of the activities for each of the programmes support available evidence on the implementation of a number of these programmes. For example, a number of studies also confirmed the implementation of Community Sexual and Reproductive Health Outreach Programmes in Ghana [44, 45]. Similarly, various studies show evidence of the implementation of programmes for reducing adolescent pregnancy in LMICs [46, 47]. The World Health Organisation provides comprehensive guidelines upon which these programmes are implemented [48]. An evaluation of 58 programmes in India by the United Nations concluded that the integration of the topic of pregnancy in life skills or sexuality education is critical in pregnancy prevention and plays a key in achieving SDG 1, 3, and 5 that aim at eliminating poverty, promoting healthier lives, and achieving gender equality respectively [49]. One of the possible reasons for this level of policy knowledge and awareness in our study could be due to the purposive sampling of participants who were directly responsible for, and had experience in, the delivery of programmes aimed to reduce adolescent pregnancy.

As part of the detailed description of the programmes, the participants also described the effectiveness of some of the programmes in reducing adolescent pregnancy in Ghana. This aligns with other literature from Ghana. For instance, between 2004 and 2011, the Time with Grandma programme was reported to have contributed to a reduction of teenage pregnancy from 15.2% to 14.3% in the Central Region of Ghana [50]. An assessment of components of Ghana’s CSE on the timing of sexual debut among in-school youth showed that CSE helped to delay sexual debut among in-school adolescent girls [51]. Such programmes are not only relevant in Ghana but can also play major roles in reducing the high prevalence of adolescent pregnancy across sub-Saharan Africa [6]. However, the paucity of available data, and the brevity of even anecdotal knowledge of policy and programme outcomes, is noteworthy. Results of a systematic review of programme implementation for adolescent girls in LMICs identified gaps in terms of the evaluation of some of these programmes [52]. Similar gaps in the evaluation of policies was also found in a scoping review of policies aimed at reducing adolescent pregnancy in Anglophone sub-Saharan Africa [18].

## Strengths and limitations

Our sampling methods allowed us to compare and contrast the knowledge and awareness of the two different groups of stakeholders who are directly involved in policy and/or programme implementation. Purposive sampling ensured that information was obtained from those who were known to have in-depth knowledge or experience about the subject and included stakeholders across three districts and eleven government and non-government organisations. Moreover, cross-checking the policies described by the health and education professionals with desktop review of these policy documents supports the credibility of the information provided by participants.

Study limitations include that some of the participants were located through the help of the Director for the Department of Gender for the Central region, which may have meant that potential participants not known to this individual were missed. These participants may also have represented a better-informed sub-set of the population. Content analysis reported in this paper is purely descriptive but more detailed findings conceptually based on implementation science are reported elsewhere [16].

## Conclusion

Our study has implications for those responsible both for the development and delivery of policy and programme interventions in LMICs. The knowledge and awareness deficits revealed in these interviews represent opportunities to promote the delivery and uptake of interventions to enhance sexual and reproductive health and reduce rates of adolescent pregnancy. The study has identified many gaps in stakeholders’ knowledge and their limited awareness of policies. Increased stakeholder engagement is required to enhance their awareness and knowledge and hence to promote implementation of these policies and programmes. Given the range of challenges mentioned by participants, future study should also explore barriers and facilitators to policy and programme implementation, and how best these may be addressed and incorporated, respectively. Finally, the inclusion of evaluation strategies may enable estimation of return on investment and motivate engagement at all levels of policy and programme delivery where benefits can be demonstrated.

### Supplementary Information

**Additional file 1. **Indepth interview guide for health/education professionals.

## Data Availability

The data for this study are available upon request.

## Abbreviations

ASRH: Adolescent sexual and reproductive health
COPNAG: Community Parents Network Advocacy Group
CSE: Comprehensive sexuality education
DOVVSU: Domestic Violence and Victim Support Unit
GW: Grassroot workers
HEP: Health education professionals
LEAP: Livelihood Empowerment against Poverty
LMICs: Low-and middle-income countries
NGOs: Non-governmental organisations
PASS: Promoting Safe Space for Adolescents
PECACEM: Promotion, Empowerment and Community Action against Child Marriage
PPAG: Planned Parenthood Association of Ghana
RHESY: Reproductive Health and Education Services for Youth or Youth people
SRH: Sexual and Reproductive Health
UNFPA: United Nations Population Fund
