# Supplementary material for: Knowledge and awareness of policies and programmes to reduce adolescent pregnancy in Ghana: a qualitative study among key stakeholders
Source: Reprod Health. 2023 Sep 22;20:143. doi: 10.1186/s12978-023-01672-2 (PMC10517459; doi:10.1186/s12978-023-01672-2)
Supplement: Supplementary file 1 — Additional file 1. Indepth interview guide for health/education professionals. [file 12978_2023_1672_MOESM1_ESM.docx]

## Indepth interview guide for health/education professionals

**Section A: Socio-demographic characteristics**

1. Age
2. Educational level
3. Years of experience
4. Role in the organisation

**Section B: Policies/programmes that have been implemented with the aim of reducing adolescent pregnancy in Ghana**

1. Policies (for policy implementers) or programmes (for programme coordinators) aimed of reducing adolescent pregnancy in Ghana?
2. Factors that influence the implementation of those policies/programmes (Probe for: child marriage, gender-based violence, early sexual initiation, multiple sexual partners, coerced sex, lack of contraceptive education, lack of affordable and adequate contraceptive commodities and inconsistent and incorrect condom use, poverty, illiteracy, unemployment and school drop-outs).
3. Activities that have been implemented to reduce adolescent pregnancy in Ghana? (Probe for: information, education and communication (IEC); advocacy; provision of adolescent reproductive health services; comprehensive sexuality education; youth empowerment; training of healthcare providers; education of parents and community members)

**Section C: Policies and programmes which have been effective or ineffective in reducing adolescent pregnancy**

1. Can you mention specific policies and programmes which you think have been effective or ineffective in reducing adolescent pregnancy in Ghana?
2. Why do you think those policies and programmes were effective or not?

**Section D: Facilitators towards the implementation, adoption, uptake and effectiveness of policies and programmes aimed at reducing adolescent pregnancy in Ghana**

1. Community factors (Probe for: knowledge of the causes of adolescent pregnancy; politics; funding)
2. Provider characteristics (Probe for: perception on the need for the policy/programme; perception on the benefits of the policy/programme; self-efficacy; skills)
3. Innovation characteristics (Probe for: adaptability and compatibility)
4. Government/organisational capacity (Probe for: government/organizational norms regarding change; integration of new policy/programme; shared vision; shared decision-making; coordination with other agencies; communication; formulation of tasks; leadership; managerial/supervisory/administrative support)
5. Training and technical assistance
6. Characteristics of adolescents (Probe for: their need for the innovation; their goals; their culture; their values).

**Section E: Barriers towards the implementation, adoption, uptake and effectiveness of policies and programmes aimed at reducing adolescent pregnancy in Ghana**

1. Community factors (Probe for: lack of knowledge of the causes of adolescent pregnancy; politics; lack of funding)
2. Provider characteristics (Probe for: perception on the need for the policy/programme; perception on the benefits of the policy/programme; self-efficacy; lack of the skills necessary for implementation)
3. Innovation characteristics (Probe for: adaptability and compatibility)
4. Government/organisational capacity (Probe for: government/organizational norms regarding change; integration of new policy/programme; shared vision; shared decision-making; coordination with other agencies; communication; formulation of tasks; leadership; managerial/supervisory/administrative support)
5. Lack of training and technical assistance
6. Characteristics of adolescents (Probe for: their need for the innovation; their goals; their culture; their values).

**Section F: Recommendations to improve the implementation of policies and programmes aimed at reducing adolescent pregnancy in Ghana**

1. What do you think can be done to improve policies and programmes and their implementation, aimed at reducing adolescent pregnancy in Ghana?

## Indepth interview guide for grassroots workers

**Section A: Socio-demographic characteristics**

1. Age
2. Educational level
3. Years of experience
4. Role in the community

**Section B: Policies/programmes that have been implemented with the aim of reducing adolescent pregnancy in Ghana**

1. Policies (for policy implementers) or programmes (for programme coordinators) aimed of reducing adolescent pregnancy in Ghana?
2. Factors that influence the implementation of those policies/programmes (Probe for: child marriage, gender-based violence, early sexual initiation, multiple sexual partners, coerced sex, lack of contraceptive education, lack of affordable and adequate contraceptive commodities and inconsistent and incorrect condom use, poverty, illiteracy, unemployment and school drop-outs).
3. Activities that have been implemented to reduce adolescent pregnancy in Ghana? (Probe for: information, education and communication (IEC); advocacy; provision of adolescent reproductive health services; comprehensive sexuality education; youth empowerment; training of healthcare providers; education of parents and community members)

**Section C: Policies and programmes which have been effective or ineffective in reducing adolescent pregnancy**

1. Can you mention specific programmes which you think have been effective or ineffective in reducing adolescent pregnancy in the central region or this community?
2. Why do you think those programmes were effective or not?

**Section D: Facilitators towards the implementation, adoption, uptake and effectiveness of policies and programmes aimed at reducing adolescent pregnancy in Ghana**

1. Community factors (Probe for: knowledge of the causes of adolescent pregnancy; politics; funding)
2. Provider characteristics (Probe for: perception on the need for the policy/programme; perception on the benefits of the policy/programme; self-efficacy; skills)
3. Innovation characteristics (Probe for: adaptability and compatibility)
4. Government/organisational capacity (Probe for: government/organizational norms regarding change; integration of new policy/programme; shared vision; shared decision-making; coordination with other agencies; communication; formulation of tasks; leadership; managerial/supervisory/administrative support)
5. Training and technical assistance
6. Characteristics of adolescents (Probe for: their need for the innovation; their goals; their culture; their values).

**Section E: Barriers towards the implementation, adoption, uptake and effectiveness of policies and programmes aimed at reducing adolescent pregnancy in Ghana**

1. Community factors (Probe for: lack of knowledge of the causes of adolescent pregnancy; politics; lack of funding)
2. Provider characteristics (Probe for: perception on the need for the policy/programme; perception on the benefits of the policy/programme; self-efficacy; lack of the skills necessary for implementation)
3. Innovation characteristics (Probe for: adaptability and compatibility)
4. Government/organisational capacity (Probe for: government/organizational norms regarding change; integration of new policy/programme; shared vision; shared decision-making; coordination with other agencies; communication; formulation of tasks; leadership; managerial/supervisory/administrative support)
5. Lack of training and technical assistance
6. Characteristics of adolescents (Probe for: their need for the innovation; their goals; their culture; their values).

**Section F: Recommendations to improve the implementation of policies and programmes aimed at reducing adolescent pregnancy in Ghana**

1. What do you think can be done to improve policies and programmes and their implementation, aimed at reducing adolescent pregnancy in Ghana?
